# Inverting the Stereoselectivity of an NADH‐Dependent Imine‐Reductase Variant

**DOI:** 10.1002/cctc.202101057

**Published:** 2021-10-22

**Authors:** Peter Stockinger, Niels Borlinghaus, Mahima Sharma, Benjamin Aberle, Gideon Grogan, Jürgen Pleiss, Bettina M. Nestl

**Affiliations:** ^1^ Institute of Biochemistry and Technical Biochemistry Department of Technical Biochemistry Universitaet Stuttgart Allmandring 31 70569 Stuttgart Germany; ^2^ York Structural Biology Laboratory Department of Chemistry University of York YO10 5DD York UK

**Keywords:** biocatalysis, imine reductase, stereoselectivity, molecular dynamics simulations, crystal structure

## Abstract

Imine reductases (IREDs) offer biocatalytic routes to chiral amines and have a natural preference for the NADPH cofactor. In previous work, we reported enzyme engineering of the (*R*)‐selective IRED from *Myxococcus stipitatus* (NADH‐IRED‐*Ms*) yielding a NADH‐dependent variant with high catalytic efficiency. However, no IRED with NADH specificity and (*S*)‐selectivity in asymmetric reductions has yet been reported. Herein, we applied semi‐rational enzyme engineering to switch the selectivity of NADH‐IRED‐*Ms*. The quintuple variant A241V/H242Y/N243D/V244Y/A245L showed reverse stereopreference in the reduction of the cyclic imine 2‐methylpyrroline compared to the wild‐type and afforded the (*S*)‐amine product with >99 % conversion and 91 % enantiomeric excess. We also report the crystal‐structures of the NADPH‐dependent (*R*)‐IRED‐*Ms* wild‐type enzyme and the NADH‐dependent NADH‐IRED‐*Ms* variant and molecular dynamics (MD) simulations to rationalize the inverted stereoselectivity of the quintuple variant.

## Graphical abstract

**Stereoselective imine reductases**: We describe the development of a NADH‐dependent *S*‐selective variant of the *Myxococcus stipitatus* imine reductase to produce useful amine products. The enantiomeric excess in the biocatalytic production was altered from up to >99 % *R*‐selectivity (parent enzyme) to up to 94 % *S*‐selectivity.

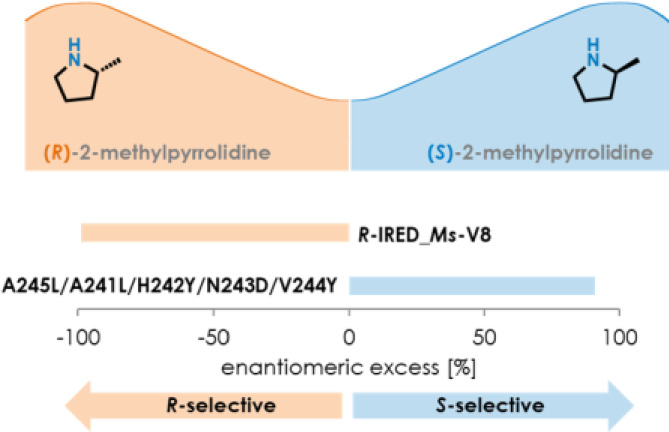

One of the most sophisticated catalytic properties of enzymes is their excellent stereoselectivity when forming chiral products from prochiral substrates. Structural analysis combined with enzyme engineering aids in understanding the molecular basis of stereoselectivity and provides tools to manipulate this property for asymmetric transformations in organic chemistry. In our ongoing efforts to develop enzymes for chiral amine synthesis, we have investigated the asymmetric reduction of prochiral imines and reductive amination with imine reductases (IREDs). The studies with the (*R*)‐selective IRED from *Myxococcus stipitatus* ((*R*)*‐*IRED‐*Ms*) demonstrated the efficient formation of various chiral nitrogen‐containing heterocycles.^[1]^ The extraordinarily broad substrate spectrum of (*R*)*‐*IRED‐*Ms* and its high activity in double reductive aminations for piperazines is so far unique. However, only (*R*)‐products are accessible with this enzyme and for some pharmaceutical building blocks, the enantiocomplementary product is desired.

Although several (*S*)‐selective IREDs have already been described,[^2^, ^3^, ^4^, ^5^, ^6^, ^7^, ^8^, ^9^, ^10^, ^11^, ^12^] no IRED with stereocomplementary selectivity and comparable substrate scope to (*R*)*‐*IRED‐*Ms* was identified. Moreover, all characterized members of the IRED family natively exhibit NADPH specificity. Cofactor regeneration systems in engineered whole‐cell systems would benefit from NADH‐dependent IREDs.[^13^, ^14^] Recently, two successful strategies were used to separately demonstrate the mutability of two (*R*)‐selective IREDs from *Streptomyces* sp. GF3587 and *Myxococcus stipitatus* ((*R*)‐IRED‐*Ms*), which switch their cofactor preference from NADPH to NADH.[^14^, ^15^, ^16^]

To date, the enzyme family of IREDs includes more than 1100 members.^[6]^ This family can be divided into two subfamilies, distinguished by their stereoselectivities; tentatively referred to as (*R*)‐ and (*S*)‐IREDs.^[17]^ A standard numbering scheme for IREDs was introduced to predict (*R*)‐ or (*S*)‐stereoselectivity by a pendant residue in the active site, D or Y, respectively, at standard position 178.[^6^, ^17^] However, this approximate classification cannot be applied to all IREDs, as different stereoselectivities have been described depending on the substrate.[^12^, ^18^] This is illustrated by recently characterized IREDs that were found to be (*S*)‐selective despite having aspartic acid as a pendant residue in the active site.[^19^, ^20^] In addition, single‐point variants of the IRED from *Amycolatopsis orientalis* displayed inverted stereoselectivity compared to the wild‐type.^[21]^ Despite extensive studies of IREDs,[^12^, ^16^, ^18^, ^22^, ^23^, ^24^, ^25^, ^26^] the molecular mechanism of asymmetric imine reduction and, if relevant, imine protonation, remains unclear.^[27]^ One mechanism has been proposed for the IRED subfamily of fungal reductive aminases (RedAms). Residues D169 and Y177 (standard positions 187 and 195) have been suggested to be involved in both protonation and deprotonation of reaction intermediates.[^21^, ^28^, ^29^] Furthermore, conformational changes in cofactor binding indicate a significantly reduced volume of the substrate‐binding site in closed enzyme‐cofactor complexes.[^10^, ^30^, ^31^] Unfortunately, the lack of ternary enzyme‐cofactor‐substrate complexes with imine substrates hinders a detailed mechanistic understanding.^[27]^ These issues are associated with highly flexible regions and domain movements that lead to conformational changes,^[32]^ as reflected in unresolved binding‐site loops and the missing nicotinamide ring of the NADPH cofactor in the ternary enzyme‐cofactor‐product complex of the IRED from *Amycolatopsis orientalis*.^[33]^

Herein, we report the iterative successive engineering of the (*R*)‐selective NADH‐variant of the IRED from *Myxococcus stipitatus* (NADH‐IRED‐*Ms*) towards inverted stereoselectivity in the asymmetric reduction of the cyclic imine 2‐methylpyrroline. We also used X‐ray structure determination and molecular dynamics (MD) simulations to rationalize the structural basis of the switched cofactor specificity and inversion in stereoselectivity, respectively.

The crystal structures of (*R*)‐IRED‐*Ms* (PDB code: 6TO4) and NADH‐IRED‐*Ms* (PDB code: 6TOE) were determined in complex with NADP^+^ and NAD^+^, respectively. The NADH‐specific variant NADH‐IRED‐*Ms* encompassed the mutations N32E/R33Y/T34E/K37R/L67I/T71V. The solved structures exhibit the canonical IRED fold^[24]^ with an N‐terminal Rossmann domain connected to a C‐terminal helical bundle *via* a long interdomain helix.

Two monomers form a dimer, with the N‐terminal domain of one monomer and the C‐terminal domain of the other forming an active‐site pocket that contain the cofactor (Figure 1A). Comparison of the (*R*)‐IRED‐*Ms* monomer with other IREDs in the PDB using the DALI server[^34^, ^35^] revealed closest‐structural homology to (*R*)‐IRED‐*Sr* from *Streptosporangium roseum* (PDB code: 5OCM;^[31]^ 36 % sequence identity; rmsd 2.1 Å over 286 Cα atoms). Analysis of the cofactor binding loop recognizing the 2’‐phosphate of NADP^+^ revealed prominent interactions between this group and the side chains of N32, R33, T34 and K37 (Figure 1B). Superposition of the structure of NADH‐IRED‐*Ms* in complex with NAD^+^ (Figure 1C) revealed changes in enzyme‐cofactor interactions. Accordingly, the carboxylate introduced at position 32 interacts with the free ribose hydroxyls of the NAD^+^ ribose. The residue Y33, which replaces arginine, is no longer in close contact with the ribose. However, it fulfils a stacking interaction function with the adenine ring system, as with R33 in the wild‐type. The lack of interaction of the new side chains of E34 and R37 with ribose hydroxyls is reflected in their mobility, resulting in poorer electron density for these side chains in the NADH‐IRED‐*Ms* NAD^+^ structure.

**Figure 1:**
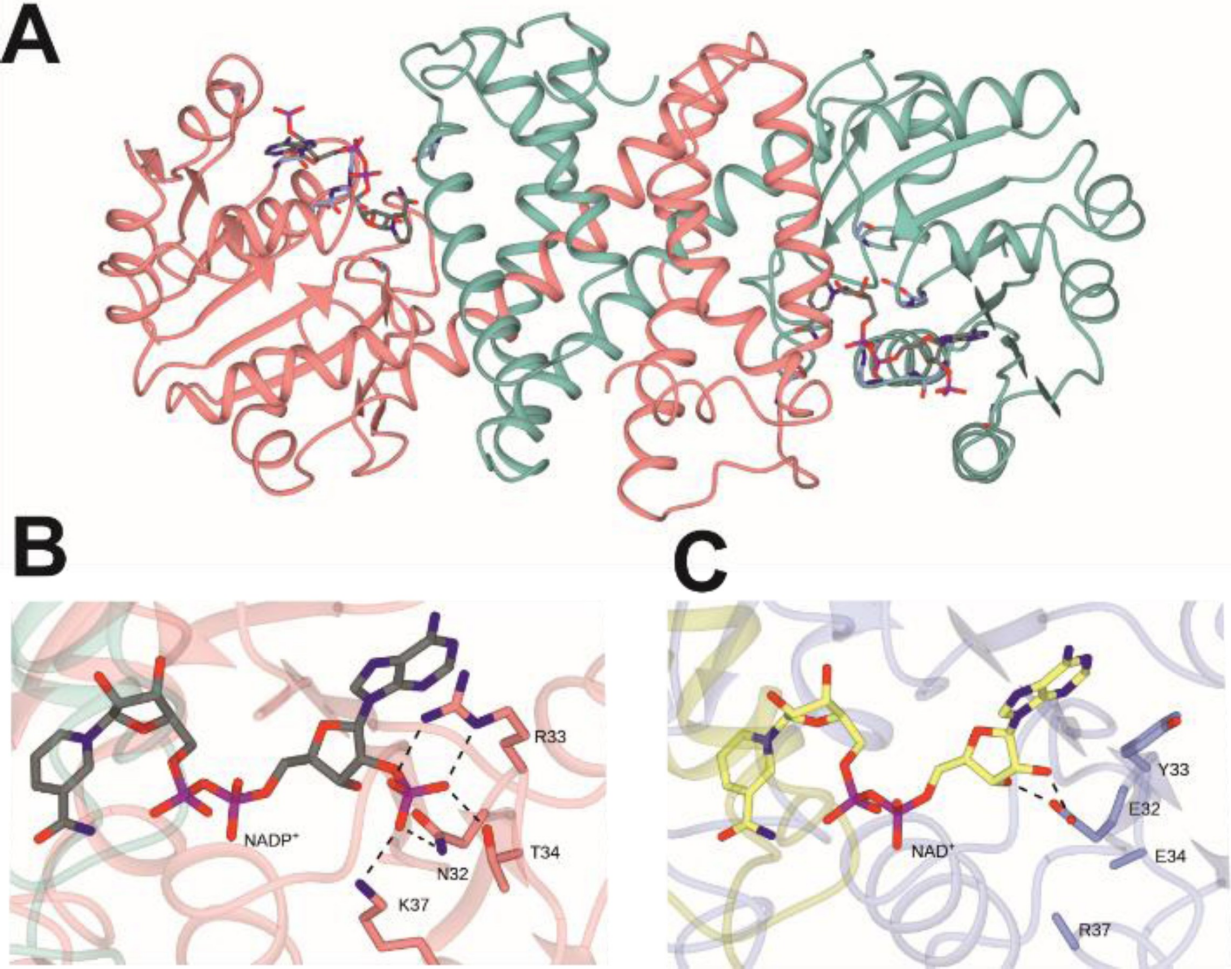
Structures of (*R*)‐IRED_*Ms* wild‐type and NADH‐IRED‐*Ms*. **A**: Dimeric structure of (*R*)‐IRED‐*Ms* in complex with NADP^+^; **B**: NADP^+^ binding in (*R*)‐IRED_*Ms*; **C**: NAD^+^ binding in NADH‐IRED‐*Ms*. Selected interactions between the cofactors and the active‐site side‐chains are indicated by black dashed lines. The side chains of E34 and R37 are absent in **C** as they could not be modelled.

To identify targets for mutagenesis, the active sites of stereocomplementary IREDs were compared to identify amino acid that mediate stereoselectivity. The best‐known residue in (*R*)‐IRED_*Ms* is D171 (standard numbering position 178), which is also described as a catalytically active amino acid. Most (*R*)‐selective IREDs have an aspartic acid at this position, in contrast to tyrosine in most (*S*)‐selective IREDs. The function of the conserved aspartic acid or tyrosine is still unclear although exchanging this residue for alanine or phenylalanine resulted in enzymes with greatly reduced activity.[^6^, ^11^, ^23^]

To switch the stereoselectivity of (*R*)‐IRED_*Ms*, D171 was first chosen as the target site for saturation mutagenesis. The resulting D171X variants (including D171Y) showed little or no catalytic activity, indicating the key role of this residue in the catalytic cycle. Similarly, the site‐saturation mutagenesis at other positions reported to be essential for (*S*)‐selectivity of IREDs did not result in altered stereoselectivity of NADH‐IRED‐*Ms* (Table S1).

We next applied site‐directed mutagenesis of (*R*)‐IRED_*Ms* to randomize the residues at positions 123, 171 and 178, which have been described in literature to play a role in determining the stereoselectivity of IREDs.[^6^, ^12^, ^17^] We performed a multiple sequence alignment of 31 different stereocomplementary IREDs (12 (*S*)‐ and 19 (*R*)‐selective IREDs, Figure S1 in the Supporting Information).

Mutant libraries were analyzed by monitoring the depletion of the NADH cofactor in 96‐well microtiter plates. Furthermore, saturation mutagenesis of V212, the equivalent position of W210 of the IRED from *Amycolatopsis orientalis*, the mutation of which has been shown to invert stereoselectivity,^[33]^ was also unsuccessful in altering this property in (*R*)‐IRED‐*Ms*. We therefore concluded that further enzyme engineering of different residues may be required to alter the enzymatic discrimination of the *re‐* and *si*‐faces of the prochiral imine.

Structural information obtained from the crystal structures of NADH‐IRED‐*Ms* in complex with NADH was used to identify amino acids near the cofactor (Table S2). Subsequent screening of (limited) site‐saturation mutagenesis libraries that included position 13, 95, 120–124, 171, 175, 178, 212, 237, 241, 242, and 245 identified the mutation A245 C, which slightly decreased the stereoselectivity. Therefore, a second mutant library was constructed by randomizing residues in the region 240–246 (standard positions 256–259) and combining them into pairs (Table S3) to account for synergistic effects. Screening revealed a double variant A241V/H242Y that formed slightly less (*R*)*‐*enantiomeric product. Finally, further exchanges (see Table S3) in the active site revealed that mutations in a specific region (A241–A245) contributed to changes in stereoselectivity (Figure 2). While a point mutation A245 L showed significant deterioration in stereoselectivity, the combination of five mutations in this region A241V/H242Y/N243D/V244Y/A245L (variant designated (*S*)‐NADH_V11) switched stereoselectivity from >99 % *ee* (*R*) to 91 % *ee* (*S*) in the reduction of 2‐methylpyrroline (**1**). Besides imine 1, three additional imines methyl‐ (**2**) and phenyl piperideine (**3**) as well as 1‐methyl‐3,4‐dihydroisoquinoline (**4**) were transformed to the corresponding amine products (**1 a‐4 a**) using the (*S*)‐NADH_V11 variant (Table 1). It is worth noting that the final variant comprising 11 mutations had reduced stability and expression levels were also poorer.

**Figure 2:**
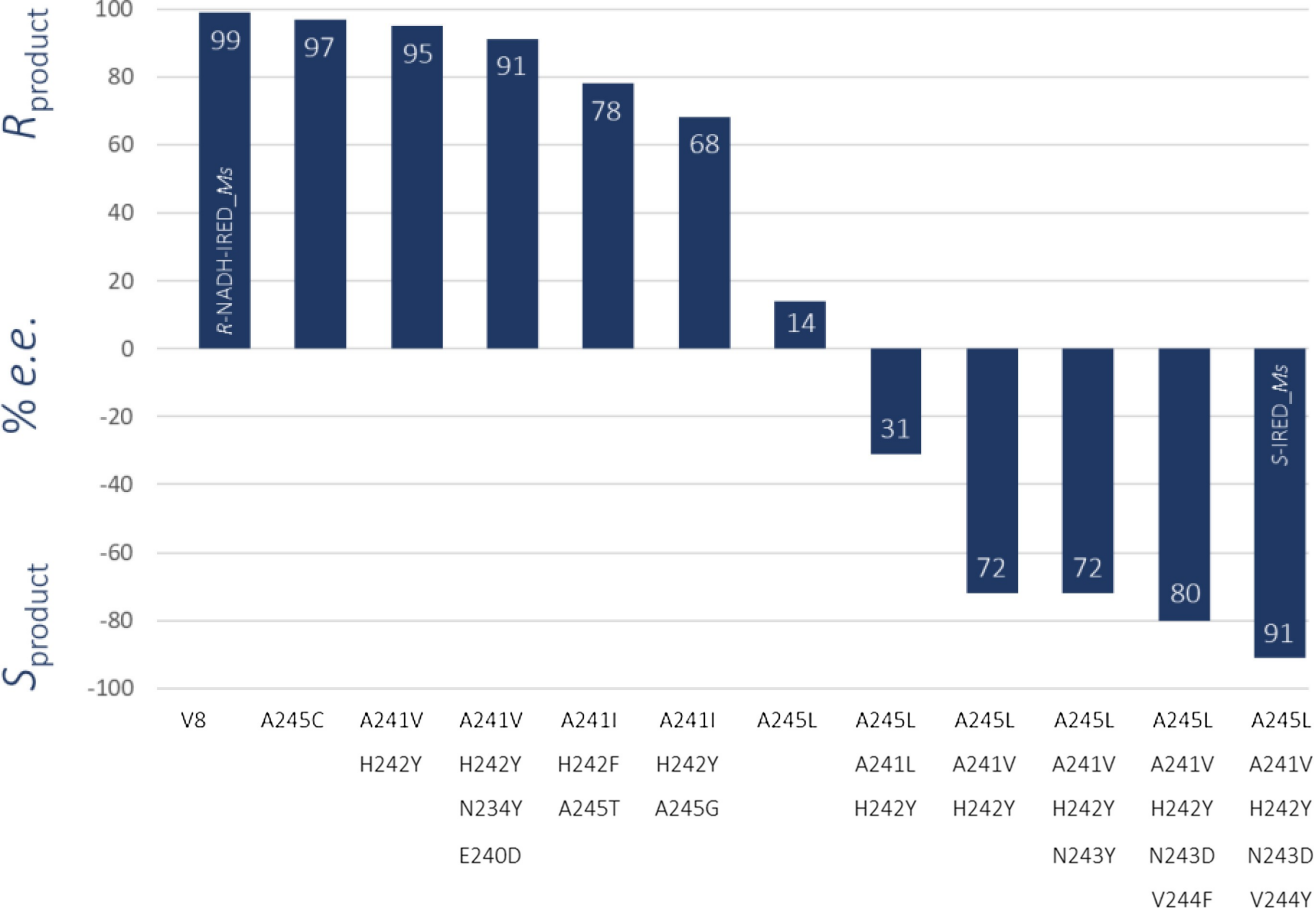
Engineering of (*S*)‐imine‐reducing selectivity for variant NADH‐IRED‐*Ms*. Enantiomeric excesses (*e.e*.) are given in percentage. NADH‐IRED‐*Ms* corresponds to the *R*‐selective NADH‐dependent IRED variant with mutations N32E/R33Y/T34E/K37R/L67I/T71V in the cofactor and substrate binding pocket that was used as parent in the engineering approach.

**Table 1:** Product formations and enantiomeric excesses in the asymmetric reduction of four model imines. For comparison, biotransformations with the NADPH‐dependent NADH‐IRED‐*Ms* variant and with the (*S*)‐selective IRED from *Paenibacillus elgii* ((*S*)‐IRED_*Pe*) were performed.

Substrate	Product	NADH‐IRED‐*Ms*	(*S*)‐NADH_V11^[a]^	(*S*)‐IRED_*Pe* ^[b]^
		Product formation [%] (*e.e*)		
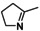	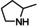	>99	>99	>97
**1**	**1 a**	(>99 % *R*)	(91 % *S*)	(95 % *S*)
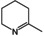	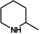	94	65	69
**2**	**2 a**	(>99 % *R*)	(94 % *S*)	(>99 % *S*)
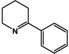	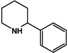	>99	77	14
**3**	**3 a**	(99 % *S*)	(94 % *R*)	(67 % *R*)
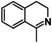	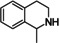	n.d.	2	78
**4**	**4 a**		(87 % *S*)	(94 % *S*)

[a] A quintuple variant of the *R*‐selective variant NADH‐IRED‐*Ms* carrying A241V/H242Y/N243D/V244Y/A245L point mutations, [b] The *S*‐selective IRED from *Paenibacillus elgii* is a natural NADPH‐dependent enzyme. n.d.=not determined

To gain insight into the molecular basis of the inverted stereoselectivity observed for NADH‐IRED‐*Ms* and (*S*)‐NADH_V11, ternary enzyme‐NADH‐substrate complexes of both variants were studied by molecular dynamics (MD) simulations. The resulting trajectories were examined for close contacts between potentially proton‐donating residues (Table S7) and the imine nitrogen atom of the substrate.

Analysis of all replicates of six molecular systems (see Supporting Information) identified three residues that interact with the substrate's imine nitrogen: S95, D171 and W179. This finding underscores the functional relevance of the previously proposed imine‐polarizing flanking residues, the conventional proton donor, and the putative alternative proton donor (IRED standard positions 112, 187, and 195, respectively).^[27]^ For all three residues, the respective stereoselectivity was assessed by classifying the orientation of the bound 2‐methylpyrroline molecule with respect to the nearest hydride of NADH (Table 2). The stereopreference of NADH‐IRED‐*Ms*_2 and (*S*)‐NADH_V11_2 evaluated by the simulations was consistent with that observed experimentally. To further investigate the molecular basis of stereopreference, the ternary enzyme‐cofactor‐substrate complex with the smallest distance between proton donor and substrate was selected for each enzyme.

**Table 2:** Mean frequency of close (<2.5 Å) imine‐nitrogen contacts with hydrogen atoms of residues in the substrate‐binding site of (*R*)‐ and (*S*)‐IRED_*Ms* variants calculated from ten replicates each 50 ns for three systems in MD simulations. The stereopreferences suggested by the binding behavior of 2‐methylpyrroline are displayed as ratio *re‐*face to *si‐*face (*re*:*si*).

	**IRED standard position**		
IRED variant	**111**	**187**	**195**
	*ratio re*:*si*		
IRED‐*Ms*	**S95**	**D171**	**W179**
NADH‐IRED‐*Ms*_1	0.005	–	0.009
	*0 : 6*		*99 : 1864*
NADH‐IRED‐*Ms_*2	0.000036	0.000052	0.084
	*0 : 1*	*20:0*	*1763 : 1452*
NADH‐IRED‐*Ms*_*3*	0.0041	–	0.025
	*190 : 509*		*1215 : 801*
(*S*)‐NADH_V11_1	–	0.032	0.019
		–	*49 : 15*
(*S*)‐NADH_V11_*2*	–	0.45	–
		*272 : 371*	
(*S*)‐NADH_V11_*3*	–	–	0.54
			*12268 : 11517*

Interestingly, the differences in stereopreference were caused by two distinct binding modes with a mirror‐image orientation of the nicotinamide group relative to the planar imine moiety (Figures 3A and B). While in NADH‐IRED‐*Ms*, the nicotinamide moiety is oriented towards the helical dimerization area, in (*S*)‐NADH_V11 it is oriented towards the Rossmann fold. The two different binding modes are caused by a rotation of a helix in the substrate‐binding site (residues 241–245) by an angle of 15 degrees. The introduced mutations trigger this structural difference in the corresponding helical region. This shifts the nicotinamide‐binding loop projecting into the binding pocket. The nicotinamide is stabilized differently in the two enzymes (Figure S6).

**Figure 3:**
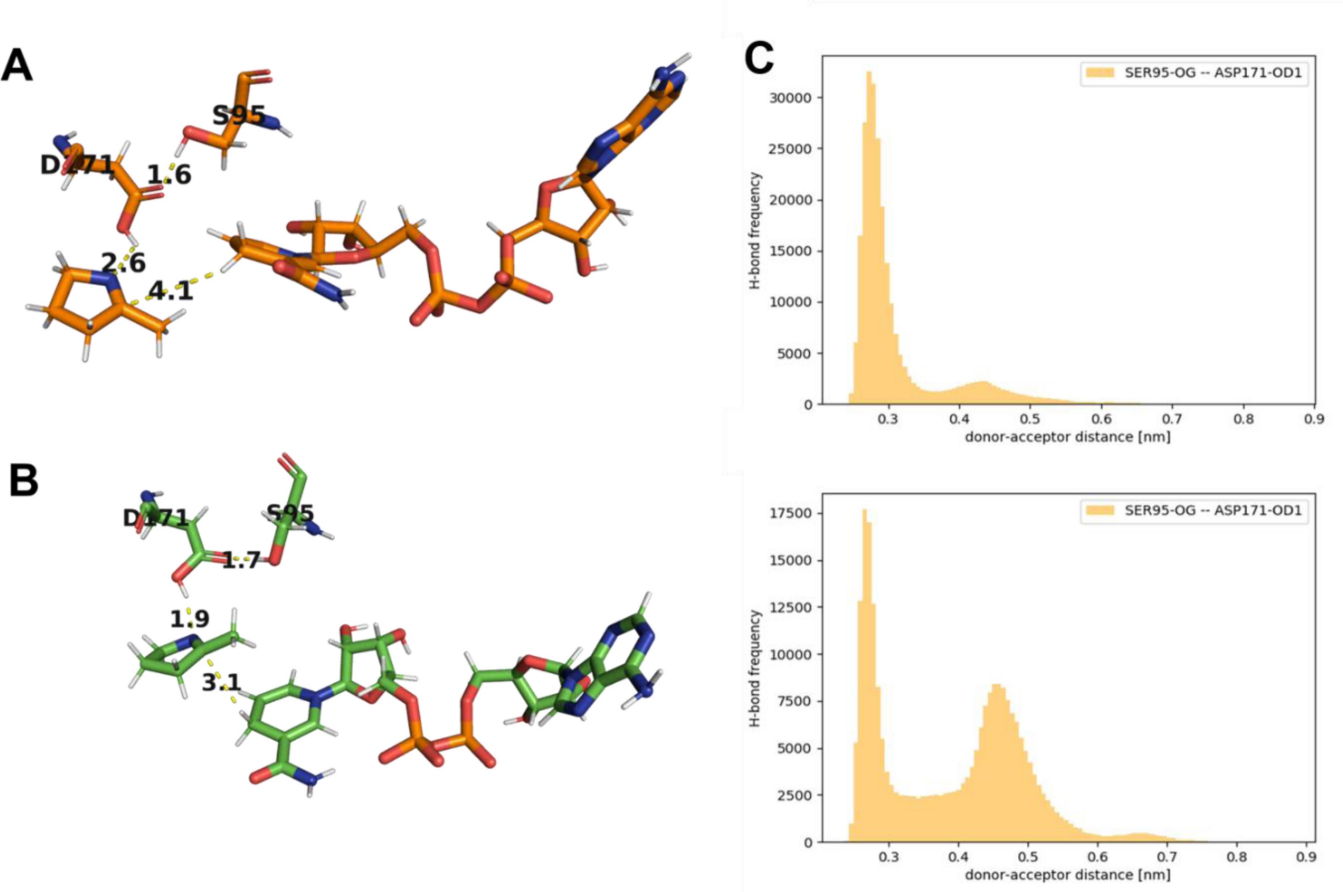
Simulation‐derived ternary complexes of (**A**) NADH‐IRED‐*Ms* and (**B**) (*S*)‐NADH_V11 with cofactor NADH and substrate 2‐methylpyrroline. H‐bonds are indicated between residues S95 and D171 (standard positions 111 and 187). Distances (yellow dashed lines) are displayed in Å. Additionally (**C**), H‐bond frequency over the donor‐acceptor distance between the putatively catalytic residues S95 and D171 were calculated for the systems (**A**) NADH‐IRED‐*Ms*_2 and (**B**) (*S*)‐NADH_V11_2.[^36^, ^37^]

Hydrogen bonding between D171 and S95 was observed in both enzymes, supporting the previously proposed functional role of ‘imine‐polarizing’ flanking‐residues.^[27]^ The higher frequency of a D171‐S95 hydrogen bond in simulation system NADH‐IRED‐*Ms*_2 compared to (*S*)‐NADH_V11_2 (Figure 3C) is consistent with the higher catalytic activity of NADH‐IRED‐*Ms*. However, it cannot be excluded that destabilizing effects of the introduced mutations cause this effect. In this context, the importance of the local electrostatic fine‐tuning, the need for proper pK_a_ matching, and the potential occurrence of proton‐relays in IREDs are highlighted.

According to these results, a catalytic dyad seems to be useful for proton donation in D‐type[^12^, ^27^] IREDs. In general, this dyad consists of an aspartic acid (standard position 187) and a flanking serine/threonine residue (standard position 111). In addition to an active role in proton donation, the passive functionality of these residues could enable the necessary protonation. In this scenario, the catalysis could occur by positioning of the C=N double bond of the imine at a polar site in the otherwise predominantly non‐polar binding pocket and by proton transfer from water molecules that are also approximated. A similar mode of operation could apply to Y‐type[^12^, ^27^] IREDs. A catalytic triad consisting of tyrosine, serine and asparagine would also provide electrostatic fine‐tuning. In particular, similar patterns in reasonable proximity to the cofactor could facilitate protonation.[^8^, ^30^]

Conversely, some residues in the large binding sites of IREDs could contribute to unproductive binding modes that limit overall catalytic efficiency. This could be the case for W179 (standard position 195) in NADH‐IRED‐*Ms* and (*S*)‐NADH_V11. The simulations performed indicate that the amine hydrogen of the indole moiety most frequently interacts with the imine nitrogen atom. Based on this observation, we speculate that this nonproductive binding mode may limit the catalytic efficiency of various IREDs. The previously described 8‐fold increased activity of variant W191 A of IR45 from *Streptomyces aurantiacus* supports this conjecture.^[38]^ However, we cannot rule out the possibility that this observation is substrate dependent or that similar mutations have a negative impact on the stereoselectivities in other IREDs. Such effects have been reported for the IRED from *Amycolatopsis orientalis*, as the Y179F and Y179 A variants resulted in inverted stereoselectivity in the reduction of multiple substrates.^[30]^ The high sequence variability, large size, and conformational flexibility of the substrate‐binding pockets of IREDs provide many degrees of freedom for electrostatic fine‐tuning of the active site. Despite this variability, IREDs exhibit remarkably high stereoselectivity. Moreover, the role of flexible regions in catalysis complicates the analysis of the determinants of stereoselectivity using X‐ray structural data.^[32]^ In contrast, MD simulations are widely used as a valuable method to analyze biochemical properties of flexible proteins on the molecular level.[^39^, ^40^] In addition, simulation‐based engineering approaches can provide the necessary time and cost efficiency for chiral amine production on an industrial scale. An example of this is the production of, for example, 1,4‐diazepanes by the pteridine reductase 1 from *Leishmania major*, an imine‐reducing member of the short‐chain dehydrogenase/reductase family.^[41]^

In summary, we demonstrate the engineering of NADH‐IRED‐*Ms* to yield a variant with high (*S*)‐selectivity and NADH specificity in the asymmetric reduction of substrate 2‐methylpyrroline. Targeted mutagenesis of a specific region in the IRED substrate‐binding pocket and the combination of mutations was key to achieving the switched selectivity. Five additional mutations in the variant NADH‐IRED‐*Ms* were sufficient to obtain a (*S*)‐selective variant with 91 % *ee*. Determining the structure of the NADH‐dependent imine reductase variant and using it for MD simulations also allowed rationalization of the altered stereopreference. Thus, the MD simulations underscore the effectiveness of this method to gain a deeper understanding of the substrate‐binding behavior of IREDs, which was previously limited by the lack of substrate complexes.

## Conflict of interest

The authors declare no conflict of interest.

## Supporting information

As a service to our authors and readers, this journal provides supporting information supplied by the authors. Such materials are peer reviewed and may be re‐organized for online delivery, but are not copy‐edited or typeset. Technical support issues arising from supporting information (other than missing files) should be addressed to the authors.

Supporting Information
