# Supplementary material for: Inverting the Stereoselectivity of an NADH‐Dependent Imine‐Reductase Variant
Source: ChemCatChem. 2021 Oct 22;13(24):5210–5. doi: 10.1002/cctc.202101057 (PMC9297850; doi:10.1002/cctc.202101057)
Supplement: Supplementary file 1 — Supporting Information [file CCTC-13-5210-s001.pdf]

ChemCatChem

Supporting Information

Inverting the Stereoselectivity of an NADH-Dependent Imine-Reductase Variant

Peter Stockinger, Niels Borlinghaus, Mahima Sharma, Benjamin Aberle, Gideon Grogan, Jürgen Pleiss,* and Bettina M. Nestl*

Content

SECTION S1. LIBRARY DESIGN AND GENERATION 2

SECTION S2. SCREENING SYSTEM. 9

SECTION S3. PROTEIN CRYSTALLIZATION; DATA COLLECTION AND REFINEMENT. 12

SECTION S4. MOLECULAR MODELLING. 16

SECTION S5. APPENDIX 20

REFERENCES. 23

Section S1. Library Design and Generation

Positions 123, 171 and 178 in NADH-IRED-*Ms* (standard positions 139, 187 and 195) were targeted as they have previously been described to be differently conserved in (*R*)- and (*S*)-selective IREDs (Figure S1).[1,2]

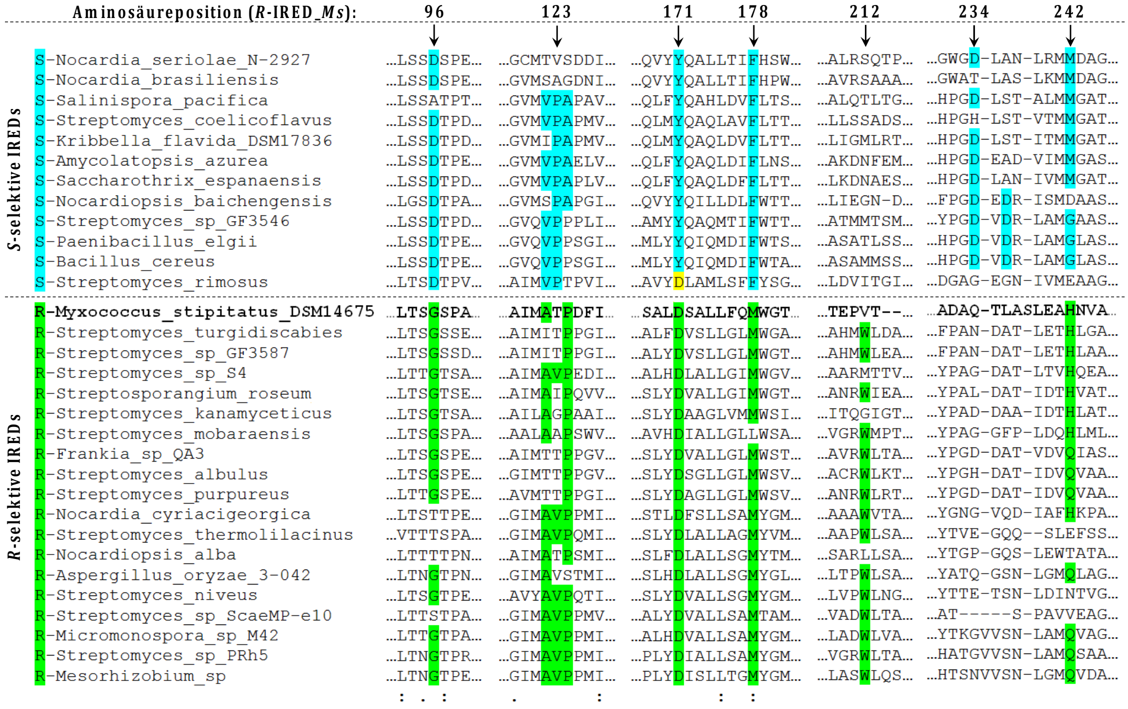

Figure S1. Multisequence alignment with 31 IREDs, 12 (*S*)-selective (blue) and 19 (*R*)-selective IREDs (green). The sequence of (*R*)-IRED_*Ms* is printed in bold. For orientation some amino acid positions are given (related to (*R*)-IRED_*Ms*). A special feature is the "D-type" (*S*)-selective IRED from *Streptomyces rimosus*. In contrast to most (*S*)-selective IREDs, it does not contain tyrosine (Y) but aspartic acid (D) at the catalytic position (marked yellow).

However, absolute rules cannot be derived from these positions. For instance, (*S*)-selective IRED from *Streptomyces rimosus*, corresponds to the "D-type", but displays (*S*)-selectivity.[2] Sequential analysis resulted in further relevant positions in the substrate binding pocket. Most (*S*)-IREDs show an aspartic acid at position 96 in NADH-IRED-Ms (standard number 112),

while (*R*)-IREDs predominantly display glycine residues at this position. Around position 123 the motif "VPA" can be found in (*S*)-IREDs while (*R*)-IREDs often display the motif "AVP". In (*R*)-IREDs, the equivalent position of residue V212 NADH-IRED-Ms (standard position 228) often displays a tryptophan, but in (*S*)-IREDs mostly small amino acids are present at this position. Interestingly, an aspartic acid at the equivalent position of Q234 (standard position 250) is conserved in (*S*)-IREDs. In contrast, no conservation was found in (*R*)-IREDs. Additionally, position 242 (standard position 257.1) was targeted, as it displays methionines or glycines in (*S*)-IREDs and histidines or glutamines in (*R*)-IREDs. Initially, several mutations were introduced and tested at the positions mentioned above. In addition, a saturation or combined partial saturation mutagenesis was carried out at positions S96 and D171 (Table S1). The reduction of 2-methylpyrroline to (*R*)- or (*S*)-2-methylpyrrolidine was selected as the model reaction. Generated enzyme variants and mutant libraries were analysed by plate screening (Section S2). However, no altered stereoselectivity was detected.

Table S1. Library design of the first mutagenesis round inspired by previously published differences in conservation described for (*R*)- and (*S*)-IREDs.

***(R)*-IRED-*Ms*_V8**	**94**	**96**	**97**	**122**	**123**	**124**	**170**	**171**	**172**	**178**	**234**	**235**	**236**	**242**
standard position	110	112	113	138	139	140	187	188	189	194	250	251	252	257.1
wildtype	T	G	S	A	T	P	L	D	S	M	Q	T	L	H
Lib1.1	T	G	S	V	P	A	L	D	S	M	Q	T	L	H
Lib1.2	T	G	S	A	T	P	L	Y	S	M	Q	T	L	H
Lib1.3	T	G	S	A	T	P	L	D	S	F	Q	T	L	H
Lib1.4	T	G	S	A	T	P	L	D	S	M	D	V	D	H
Lib1.5	T	G	S	A	T	P	L	D	S	M	Q	T	L	G
Lib1.6	T	G	S	A	T	P	X	Y	Q	M	Q	T	L	H
Lib1.7	T	D	X	A	T	P	L	D	S	M	Q	T	L	H
Lib1.8	X*	D	X*	A	T	P	L	D	S	M	Q	T	L	H

	Site-directed mutation
X*	partial saturation mutagenesis with X = A,D,F,S,V,Y
X	partial saturation mutagenesis with X = all amino acids

In the next round, additional libraries were generated in respect to the structural information provided by the crystal structure (Section S3). All amino acid positions in proximity to the C4 atom of NADH (position of the transferred hydride) were considered (Figure S2). Neighbouring positions were combined into pairs and exchanged simultaneously in a combined partial saturation mutagenesis thus partly considering combinatorial effects. A saturation mutagenesis was performed at the remaining positions (Table S2).

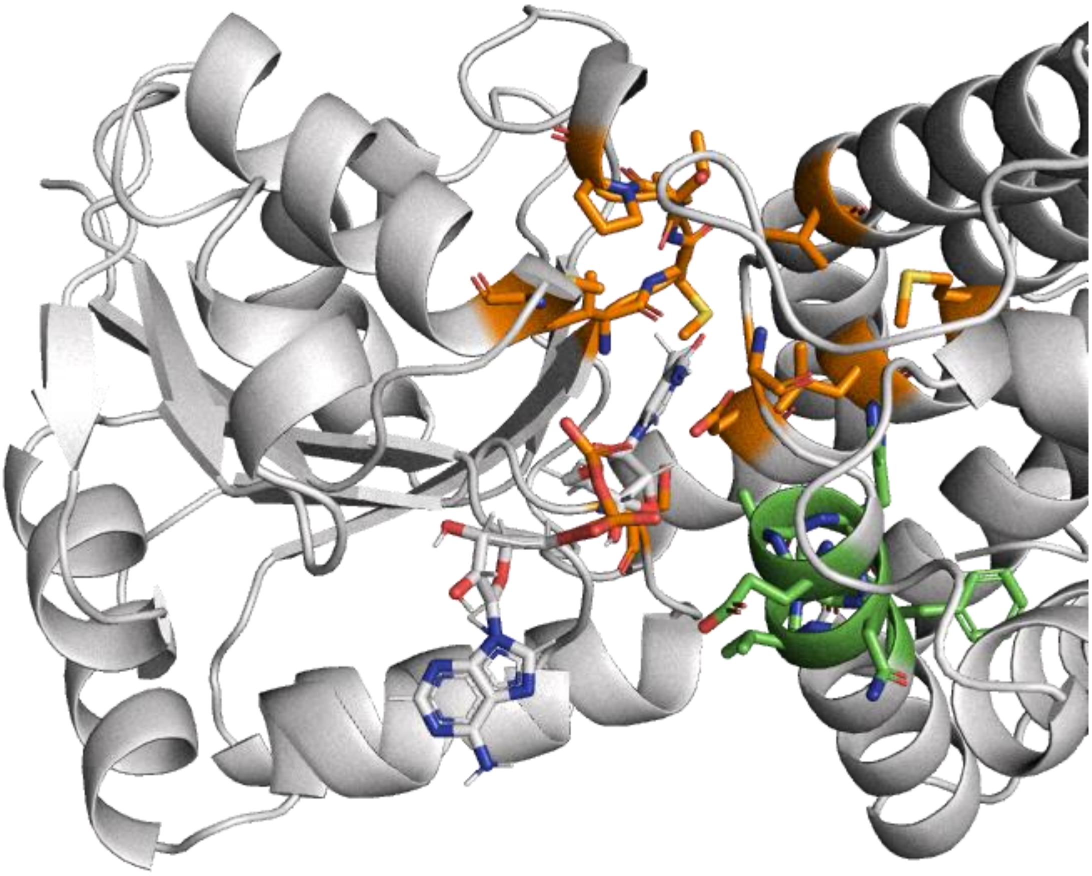

Figure S2. Residues considered for mutagenesis in the design of the second (orange) and the third (green) library.

Table S2. Library design of the second mutagenesis round focussing on residues derived using the crystal structure to identify residues in proximity to NADH C4 atom.

***(R)*-IRED-*Ms*_V8**	**13**	**95**	**120**	**121**	**122**	**123**	**124**	**171**	**175**	**178**	**212**	**237**	**241**	**242**	**245**
standard position	30	111	136	137	138	139	140	187	191	194	228	253	257	257.1	258
wildtype	M	S	I	M	A	T	P	D	L	M	V	A	A	H	A
Lib2.1	X	S	I	M	A	T	P	D	L	M	V	A	A	H	A
Lib2.2	M	X*	I	M	A	T	P	X*	L	M	V	A	A	H	A
Lib2.3	M	S	X*	X*	A	T	P	D	L	M	V	A	A	H	A
Lib2.4	M	S	I	M	X*	X*	P	D	L	M	V	A	A	H	A
Lib2.5	M	S	I	M	A	T	X	D	L	M	V	A	A	H	A
Lib2.6	M	S	I	M	A	T	P	D	X*	X*	V	A	A	H	A
Lib2.7	M	S	I	M	A	T	P	D	L	M	X	A	A	H	A
Lib2.8	M	S	I	M	A	T	P	D	L	M	V	X	A	H	A
Lib2.9	M	S	I	M	A	T	P	D	L	M	V	A	X*	X*	A
Lib2.10	M	S	I	M	A	T	P	D	L	M	V	A	A	H	X

X* partial saturation mutagenesis with X = A,D,F,S,V,Y

X partial saturation mutagenesis with X = all amino acids

Interestingly, variant A241V/H242Y in library Lib2.9 resulted in a slightly decreased enantiomeric excess (*ee* from > 99% (*R*) to 95% (*R*)). Similarly did mutation A245C in library Lib2.10 (*ee* from > 99% (*R*) to 97% (*R*)). However, variant A245L in library Lib2.10 was most promising, which displayed a significantly lower conversion but generated an almost racemic product mixture (*ee* ≈ 14% (*R*)). Based on these results, positions 241, 242 and 245, as well as the surrounding amino acids were modified in another round of mutagenesis (Table S3).

Table S3. Library design of the third mutagenesis round focussing on mutations in the helical region identified within the second mutagenesis round.

(R)-IRED-*Ms*_V8	240	241	242	243	244	245	246
standard position	256	257	257.1	257.2	257.3	258	259
wildtype	E	A	H	N	V	A	F
Lib3.1	E	V	Y	N	V	X	F
Lib3.2	E	I	Y	N	V	X	F
Lib3.3	E	X*	X*	N	V	L	F
Lib3.4	E	X	Y	N	V	A	F
Lib3.5	E	V	X	N	V	A	F
Lib3.6	E	M	X	N	V	A	F
Lib3.7	X*	V	Y	X*	V	A	F
Lib3.8	E	A	H	N	X*	C	X*
Lib3.9	E	V	Y	N	X*	C	X*
Lib3.10	E	A	H	N	X*	L	X*
Lib3.11	E	I	Y	N	X*	L	X*
Lib3.12	E	A	H	N	V	L	X
Lib3.13	E	X	Y	N	V	L	F
Lib3.14	E	V	X	N	V	L	F
Lib3.15	E	V	Y	X	V	L	F
Lib3.16	E	V	Y	X*	X*	L	F

	Site-directed mutation
X*	partial saturation mutagenesis with X = A,D,F,S,V,Y
X	partial saturation mutagenesis with X = all amino acids

As described previously,[3] substrate binding site variants were generated from pBAD33_NADH-IRED-*Ms* plasmid according to chosen screening libraries and mutagenesis methods (Section S1).

Cloning, protein expression, and purification

The variant NADH-IRED-Ms was cloned into the pBAD33 plasmid using a Gibson Assembly and fused with an N-terminal his6-tag. For the expression, we used *E. coli* JW5510 cells harboring the vector and let them grow as preculture overnight at 37 °C. Next, we inoculated terrific broth (TB) culture medium containing 34 $\mu g\,mL^{-1}$ chloramphenicol with 0.25 % preculture. After a 2–3 h incubation at 37 °C and reaching an optical density of OD_{600}=0.8–1.0, the protein expression was induced with the addition of arabinose (final concentration 0.02 %). Then, the cultures were incubated for about 20 h at 25 °C. Cells were harvested and then lysed with a high-pressure homogenizer. Protein purification was performed with cobalt His-Trap columns (His-GraviTrap-TALON, Healthcare) using buffer A (50 mm potassium phosphate buffer pH 7.0, 300 mm KCl) for binding, buffer B (50 mm potassium phosphate buffer pH 7.0, 300 mm KCl, 5 mm imidazole) for washing, and buffer C (50 mm potassium phosphate buffer pH 7.0, 300 mm KCl, 500 mm imidazole) for eluting the enzyme. Afterwards, the buffer was changed by dialysis (two times, 2 h in 5 L, 50 mm potassium phosphate buffer pH 7.0, MWCO=6–8 kDa). Purity and size were verified by SDS-PAGE. The protein concentration was determined by using the BCA Protein Assay Kit (Thermo Scientific).

Table S4. Plasmid constructs of the reported IRED variants using pBAD33.

ID	Construct	Variant
pITB5584	pBAD33_*R*-IRED-*Ms*	(*R*)-IRED-*Ms*
pITB5585	pBAD33_NADH-IRED-Ms	NADH-IRED-Ms mutations: N32E, R33Y, T34E, K37R, L67I, T71V
pITB5589	pBAD33_*S*-IRED_V11	(*S*)-IRED_V11<br>mutations: N32E, R33Y, T34E, K37R, L67I, T71V + A241V, H242Y, N243D, V244Y, A245L

All primers (Table S2) for the introduction of the mutations targeting the switch in stereoselectivity were obtained from Metabion International AG (Planegg, DE).

Table S5. Primers used to generate (*S*)-NADH_V11 variants to provoke a switched stereoselectivity. The mutation site in the primer is marked in grey.

Mutations	Template	Strategy	Method	Primer (5‘->3‘): fw = forward, rv = reverse
A122V,<br>T123P,<br>P124A	NADH_*Ms*	1	A	fw: CGGTGCGATCATG GTT CCG GCG GATTTTATTGGCCAG<br>rv: CTGGCCAATAAAATC CGC CGG AAC CATGATCGCACCG
D171Y	NADH_*Ms*	1	A	V: CATGCCTCAGCACTG TAT AGCGCCCTGCTGTTTCAG<br>R: CTGAAACAGCAGGGCGCT ATA CAGTGCTGAGGCATG
M178F	NADH_*Ms*	1	A	V: CCTGCTGTTTCAG TTC TGGGGCACCCTGTTC<br>R: GAACAGGGTGCCCCA GAA CTGAAACAGCAGG
Q234D,<br>T235V,<br>L236D	NADH_*Ms*	1	A	V: CTGACCGCAGACGCT GAT GTG GAT GCAAGTCTGGAAGG<br>R: CCTTCCAGACTTGC ATC CAC ATC AGCGTCTGCGGTCAG
H242G	NADH_*Ms*	1	A	V: CAAGTCTGGAAGCT GGT AACGTGGCGTTC<br>R: GAACGCCACGTT ACC AGCTTCCAGACTTG
S95X,<br>D171X	NADH_*Ms*	3	B	V: CAGCTGACC KHT GGTTCTCCGGCACTGGC<br>R: CAGGGCGCT ADM CAGTGCTGAGGCATGACC<br>V: CTCAGCACTG KHT AGCGCCCTGCTGTTTCAG<br>R: CGGAGAACC ADM GGTCAGCTGAACCAGCAG
I120X,<br>M121X	NADH_*Ms*	3	A	V: CTGGACGGTGCG KHT KHT GCCACCCCGGATTTTATTG<br>R: CAATAAAATCCGGGGTGGC ADM ADM CGCACCGTCCAG
A122X,<br>T123X	NADH_*Ms*	3	A	V: GACGGTGCGATCATG KHT KHT CCGGATTTTATTGGC<br>R: GCCAATAAAATCCGG ADM ADM CATGATCGCACCGTC
L175X,<br>M178X	NADH_*Ms*	3	A	V: GACAGCGCCCTG KHC TTTCAG KHT TGGGGCACCCTG<br>R: CAGGGTGCCCCA ADM CTGAAA GDM CAGGGCGCTGTC
A241X,<br>H242X	NADH_*Ms*	3	A	V: CTGGCAAGTCTGGAA KHT KHT AACGTGGCGTTCC<br>R: GGAACGCCACGTT ADM ADM TTCCAGACTTGCCAG
V212X	NADH_*Ms*	2	A	V: CTGACCGAACCG NNK ACCCAGGGTGCCGTTG<br>R: CGGCACCCTGGGT MNN CGGTTCGGTCAGTTTG
A237X	NADH_*Ms*	2	A	V: GACGCTCAGACGCTG NNK AGTCTGGAAGCTCATAAC<br>R: GAGCTTCCAGACT MNN CAGCGTCTGAGCGTCTG
M13X	NADH_*Ms*	2	A	V: GTTATTGGCGCTGGCCGT NNK GGCTCCGCACTG<br>R: CAGTGCGGAGCC MNN ACGGCCAGCGCCAATAAC
P124X	NADH_*Ms*	2	A	V: CGATCATGGCCACC NNK GATTTTATTGGCCAGGC<br>R: GCCTGGCCAATAAAATC MNN GGTGGCCATGATCG
A245X	NADH_*Ms*	2	A	V: GGAAGCTCATAACGTG NNK TTCCAACACCTGCTG<br>R: CAGCAGGTGTTGGAA MNN CACGTTATGAGCTTCC
L170X,<br>D171Y,<br>S172Q	NADH_*Ms*	1+2	A	V: CATGCCTCAGCACTG NNK TAC CAG GCCCTGCTGTTTCAG<br>R: CTGAAACAGCAGGGC CTG GTA MNN CAGTGCTGAGGCATG
A245X	15B7	2	A	V: GGAAGTTTATAACGTG NNK TTCCAACACCTGCTG<br>R: CAGCAGGTGTTGGAA MNN CACGTTCAAATATTCC
A241X,<br>H242X	110D2	3	A	V: CTGGCAAGTCTGGAA KHT KHT AACGTGTTGTTCC<br>GGAACAACACGTT ADM ADM TTCCAGACTTGCCAG
A241X	15B7	2	C	V: GCTCAGACGCTGGCAAGTCTGGAA NDT<br>TATAACGTGGCGTTCCAACACCTGC<br>V: GCTCAGACGCTGGCAAGTCTGGAA VHG<br>TATAACGTGGCGTTCCAACACCTGC<br>V: GCTCAGACGCTGGCAAGTCTGGAA TGG<br>TATAACGTGGCGTTCCAACACCTGC<br>R: CAGACTTGCCAGCGTCTGAGCGTCTGCGGTCAGGCGATTTTGCTG
H242X	15B7	2	C	V: GCTCAGACGCTGGCAAGTCTGGAAGTT NDT<br>AACGTGGCGTTCCAACACCTGCTG<br>V: GCTCAGACGCTGGCAAGTCTGGAAGTT VHG<br>AACGTGGCGTTCCAACACCTGCTG<br>V: GCTCAGACGCTGGCAAGTCTGGAAGTT TGG<br>AACGTGGCGTTCCAACACCTGCTG<br>R: CAGACTTGCCAGCGTCTGAGCGTCTGCGGTCAGGCGATTTTGCTG
A241M,<br>H242X	NADH_*Ms*	1+2	C	V: GCTCAGACGCTGGCAAGTCTGGAA ATG NDT<br>AACGTGGCGTTCCAACACCTGCTG<br>V: GCTCAGACGCTGGCAAGTCTGGAA ATG VHG<br>AACGTGGCGTTCCAACACCTGCTG<br>V: GCTCAGACGCTGGCAAGTCTGGAA ATG TGG<br>AACGTGGCGTTCCAACACCTGCTG<br>R: CAGACTTGCCAGCGTCTGAGCGTCTGCGGTCAGGCGATTTTGCTG
E240X,<br>N243X	15B7	3	B	V: GCTCAGACGCTGGCAAGTCTG KHT GTTTAT KHT<br>GTGGCGTTCCAACACCTGCTGGCC<br>R: CAGACTTGCCAGCGTCTGAGCGTCTGCGGTCAGGCGATTTTGCTG
V244X,<br>F246X	110C2	3	A	V: GTCTGGAAGCTCATAAC KHT TGT KHT CAACACCTGCTGGCC<br>R: GGCCAGCAGGTGTTG ADM ACA ADM GTTATGAGCTTCCAGAC
V244X,<br>A245C,	15B7	1+3	A	V: GTCTGGAAGTTTATAAC KHT TGT KHT CAACACCTGCTGGCC<br>R: GGCCAGCAGGTGTTG ADM ACA ADM GTTATAAACTTCCAGAC

F246X				
V244X, F246X	110D2	3	A	V: GTCTGGAAGCTCATAAC KHT TTG KHT CAACACCTGCTGGCC<br>R: GGCCAGCAGGTGTTG ADM CAA ADM GTTATGAGCTTCCAGAC
V244X, A245L, F246X	24E4	1+3	A	V: GTCTGGAAATTTATAAC KHT TTG KHT CAACACCTGCTGGCC<br>R: GGCCAGCAGGTGTTG ADM CAA ADM GTTATAAATTTCCAGAC
A245L	D171Y	1	A	V: GGAAGCTCATAACGTG TTG TTCCAACACCTGCTGG<br>R: CCAGCAGGTGTTGGAA CAA CACGTTATGAGCTTCC
A245X	24E4	2	A	V: GTCTGGAAATTTATAACGTG NNK TTCCAACACCTGCTGG<br>R: CCAGCAGGTGTTGGAA MNN CACGTTATAAATTTCCAGAC
G96D, S79X	NADH_*Ms*	1+2	A	V: GTTCAGCTGACCAGC GAT NNK CCGGCACTGGCTC<br>R: GAGCCAGTGCCGG MNN ATC GCTGGTCAGCTGAAC
T94X, G96D, S79X	NADH_*Ms*	1+3	A	V: CTGGTTCAGCTG KHT AGCGAT KHT CCGGCACTGGCTG<br>R: CAGCCAGTGCCGG ADM ATCGCT ADM CAGCTGAACCAG
F246X,	110D2	2	A	V: GAAGCTCATAACGTG TTG NNK CAACACCTGCTGGCC<br>R: GGCCAGCAGGTGTTG MNN CAA CACGTTATGAGCTTC
A241X	23B3	2	A	V: GCTGGCAAGTCTGGAA NNK TATAACGTGTTGTTCC R:<br>GGAACAACACGTTATA MNN TTCCAGACTTGCCAGC
H242X	23B3	2	A	V: GGCAAGTCTGGAAGTT NNK AACGTGTTGTTCCAAC<br>R: GTTGGAACAACACGTT MNN AACTTCCAGACTTGCC
N243X	23B3	2	A	V: CAAGTCTGGAAGTTTAT NNK GTGTTGTTCCAACACC<br>R: GGTGTTGGAACAACAC MNN ATAAACTTCCAGACTTG
N243X, V244X	23B3	3	A	V: CAAGTCTGGAAGTTTAT KHT KHT TTGTTCCAACACCTGC<br>R: GCAGGTGTTGGAACAA ADM ADM ATAAACTTCCAGACTTG

[a] Selected mutagenesis strategies:
1) single point mutations; 2) single-position saturation mutagenesis; 3) combinatorial partial saturation mutagenesis
[b] Methods to introduce the mutations:
A) QuikChange™-PCR;[5] B) *Gibson assembly*,[6] C) *Gibson assembly* combined with „22c-trick".[7]

Section S2. Screening System.

The screening system for the identification of IRED variants with altered stereoselectivity in the 96-well format was performed using gas chromatography (GC) and a chiral column. 2-Methylpyrroline was selected as model substrate for the determination of stereoselectivity. Initially, 96-well deep-well plates (Ritter riplate SW, 2 mL, Ritter GmbH, Schwabmünchen, DE) were filled with TB medium (600 µL pro well, 34 µg mL^{-1} chloramphenicol) and inoculated with single colonies of the mutant libraries. This preculture plate was incubated at 37°C and 320 rpm for about 8 h (incubation shaker HT Minitron, Infors AG, Bottmingen, CH). The preculture plate was then used to inoculate an expression plate (also a 96 deep-well plate). For this purpose, 100 µl of the preculture were added to 900 µl TB medium each (34 µg mL^{-1} chloramphenicol, 0.022% *w/v* arabinose). The expression of the (*R*)-IRED_*Ms* variants was performed at 25°C and 800 rpm for 22 h (TiMix Control with TH30 thermostat, Edmund Bühler GmbH, Hechingen, DE). Subsequently, the cells were harvested by centrifugation (4°C, 15 min, 3220 g, centrifuge: 5810 R, rotor: A-4-62, Eppendorf AG, Hamburg, DE) and the cell pellets were frozen overnight at -80°C. For enzymatic cell disruption, the pellets were resuspended

with 400 µl lysis buffer (50 mM Kpi buffer, pH 7.0, 400 µg mL^{-1} lysozyme, 5 µg mL^{-1} DNAseI) and incubated for 60 min at 37°C and 180 rpm (incubation shaker Multitron/Pro, Infors AG, Bottmingen, CH). To separate the insoluble components, the deep-well plate was centrifuged for 30 min (4°C, 3220 g, centrifuge: 5810 R, rotor: A-4-62, Eppendorf AG, Hamburg, DE). One reaction plate was prepared during each centrifugation. After centrifugation, 150 µl of the supernatant was added to the reaction plate, which had previously been mixed with 50 µl reaction mix.

Biotransformation and sample preparation

The biotransformations of imines **1-4** were carried out for 4 h at 25°C and 180 rpm (incubation shaker Multitron/Pro, Infors AG, Bottmingen, CH). After 4 h the biotransformation was stopped by adding 20 µl NaOH (5M). Derivatization and extraction followed by addition of 250 µl derivatization mix (MTBE, 4 µl mL-1 acetic anhydride). As the available sealing devices for deep-well plates were not sufficiently tight to withstand the vapour pressure when shaking MTBE, no shaking was used. Instead, the phases were mixed by pipetting up and down (10 times with 400 µl). Then 50 µl 4% aqueous methylamine solution was added to quench the excess acetic anhydride. The organic phase was then increased by adding 750 µl MTBE. Again, the phases were mixed with the pipette (8 times with 1000 µl). The phases were then separated by centrifugation (4°C, 4 min, 3220 g, centrifuge: 5810 R, rotor: A-4-62, Eppendorf AG, Hamburg, DE). 200 µl of the organic phase (top) was transferred into GC glass vessels with glass insert and sealed gas-tight. The separation and analysis of the formed product enantiomers was performed with chiral GC as described previously (Figure S3 and S4).[3,4]

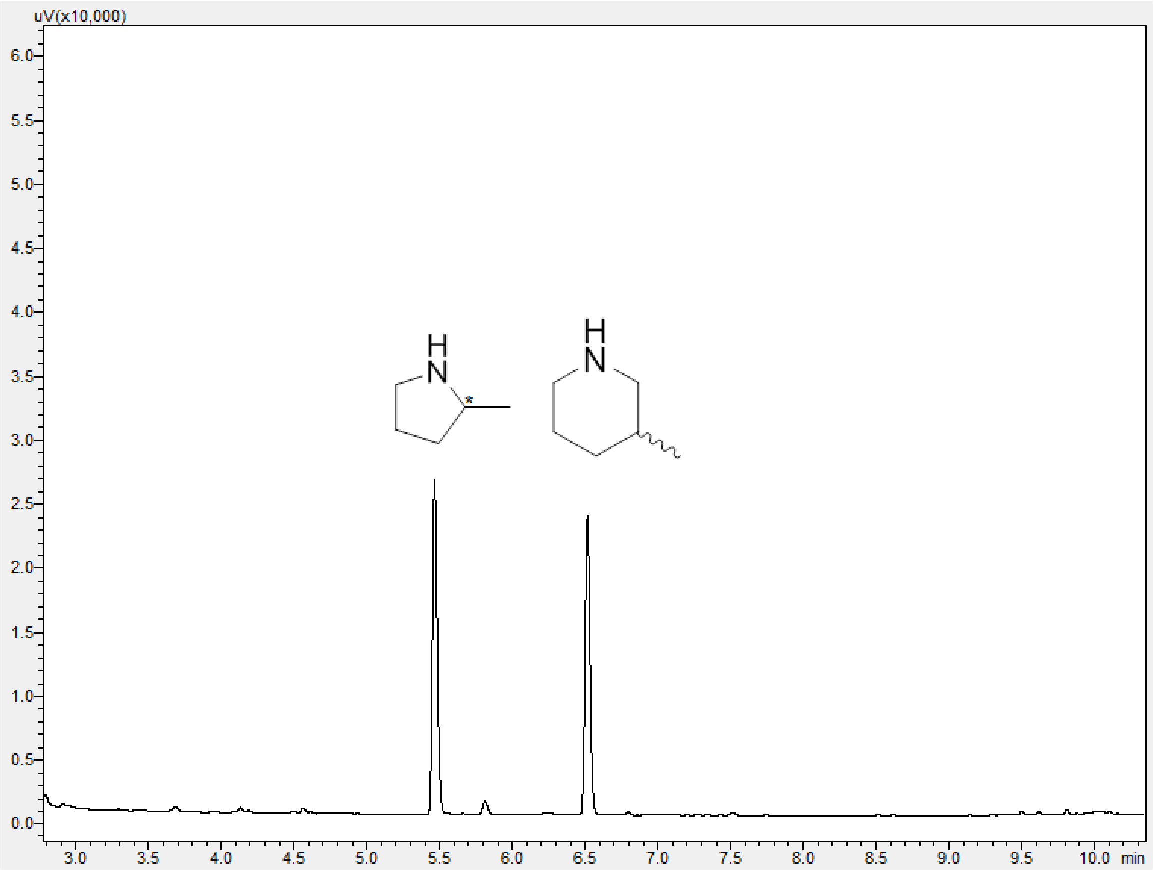

Figure S3. Chromatogram of GC analysis of reduction product 2-methylpyrrolidine (left peak) with internal standard (3-methyl piperidine, right peak).

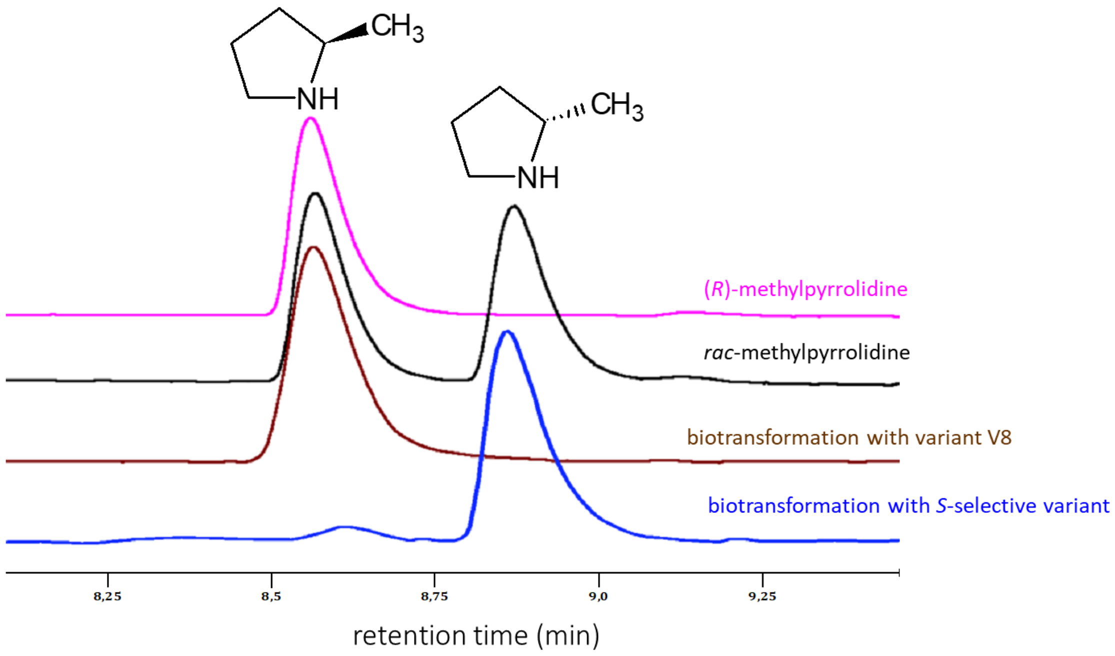

Figure S4. Example chromatogram of chiral GC measurement for the separation of (*R*)- and (*S*)-methylpyrrolidine. Beside the product standards (pink, black), the formed product enantiomers from the biotransformation with NADH-IRED-Ms (brown) or with (*R*)-IRED_*Ms*-quintuple variant ((*S*)-NADH_V11, blue) are shown.

Section S3. Protein Crystallization; Data Collection and Refinement.

Protein Crystallization

Initial screening of crystallization conditions was performed using commercially available INDEX (Hampton Research), PACT premier and CSSI/II (Molecular Dimensions) screens in 96-well sitting drop trays. Further optimization was carried out in a 24-well hanging-drop format to obtain optimized crystals for X-ray diffraction. For co-crystallization experiments, a 0.2 M stock solutions of cofactor $NADP^+$ and NAD^+ were prepared in water and 1 M stock solution of cyclohexanone was prepared in DMSO. Crystals of were grown using (*R*)-IRED-*Ms* at 34 mg mL^{-1} in 50 mM Tris pH 7.5, 300 mM NaCl in a drop containing 0.15 µL protein: 0.15 µL mother liquor comprising 0.2 M ammonium acetate, 25% PEG (polyethylene glycol) 3350 *w/v*, 0.1 M Tris pH 8.5, 5 mM $NADP^+$ as cofactor and 5 mM cyclohexanone as substrate,

however, electron density for that substrate was not observed in the final structure. Crystals of the NADH-IRED-Ms NAD^+ complex were obtained using NADH-IRED-Ms at 40 mg mL^{-1} in 50 mM Tris pH 7.5, 300 mM NaCl co-crystallized with 5 mM NAD^+ cofactor in a sitting drop containing 0.15 μL protein: 0.15 μL mother liquor, the latter comprising 20% PEG 6000 *w/v*, 0.2 M NH_4Cl in 0.1 M HEPES pH 7. The (*R*)-IRED-*Ms*-$NADP^+$ crystals were harvested directly into liquid nitrogen, using nylon CryoLoops™ (Hampton Research) using the mother liquor without any further cryoprotectant. For the NADH-IRED-Ms NAD^+ complex, 10% ethylene glycol in the mother liquor was used as a cryoprotectant.

Data Collection, Structure Solution and Refinement

The datasets described in this report were collected at the Diamond Light Source, Didcot, Oxfordshire, U.K. on beamlines I03 ((*R*)-IRED-*Ms*-$NADP^+$) and I04-1 (NADH-IRED-Ms NAD^+). Data were processed and integrated using XDS[8] and scaled using SCALA[9] included in the Xia2 processing system.[10] Data collection statistics are provided in Table S6. Crystals of wt-MsIRED-$NADP^+$ were obtained in space group $P2_1$, with four molecules in the asymmetric unit; crystals of the NADH-IRED-Ms NAD^+ complex were in space group $P6_5$. The solvent content in the crystals was 49% and 60% respectively. The structure of the wt-MsIRED-$NADP^+$ was solved by molecular replacement using MOLREP[11,12] with the monomer of *At*RedAm (PDB code 6EOD[13]) as the model. The structure was built and refined using iterative cycles in Coot[14] and REFMAC,[15] employing local NCS restraints in the refinement cycles. The structure of NADH-IRED-Ms NAD^+ was solved using the wild-type structure as the model. Following building and refinement of the protein and water molecules in this complex, residual density was observed in the omit maps at the dimer interfaces, which could be clearly modelled as $NADP^+$ and NAD^+ in the respective complexes. The final structures exhibited % R_{cryst} /R_{free} values of 21.2/23.9 and 19.2/22.8 respectively. Refinement statistics for the structures are presented in Table S6**.** The Ramachandran plot for (*R*)-IRED-*Ms*-$NADP^+$ showed 96.0% of residues to be situated in the most favoured regions, 3.5% in additional allowed and 0.5% residues in outlier regions. The values for the NADH-IRED-Ms NAD^+ complex were 95.4%, 4.5% and 0.1% respectively. The structures have been deposited in the Protein Databank (PDB) with accession codes **6TO4** ((*R*)-IRED-*Ms*-$NADP^+$ complex) and **6TOE** (NADH-IRED-Ms NAD^+ complex)**.**

Table S6. Data Collection and Refinement Statistics for wt-*Ms*IRED in complex with $NADP^+$ and NAD^+ variant in complex with NAD^+. Numbers in brackets refer to data for highest resolution shells.

	***Ms*IRED-NADP⁺**	***Ms*IRED variant NAD⁺**
Beamline	I03	I04-1
Wavelength (Å)	0.97625	0.91587
Resolution (Å)	49.27-2.29 (2.36-2.29)	48.97-2.78 (2.86-2.78)
Space Group	$P2_1$	$P6_5$
Unit cell (Å)	a = 118.71 b = 42.58; c = 121.40<br>α = γ = 90.0° β = 110.4 °	a = b = 199.45; c = 97.94<br>α = γ = 90.0° β = 120.0°
No. of molecules in the asymmetric unit	4	6
Unique reflections	52197 (4505)	55975 (4545)
Completeness (%)	100.0 (100.0)	100.0 (100.0)
R_{merge} (%)	0.09 (0.83)	0.17 (0.91)
$R_{p.i.m.}$	0.03 (0.28)	0.05 (0.29)
Multiplicity	18.1 (18.4)	20.4 (19.9)
$<I/\sigma(I)>$	20.9 (3.4)	16.1 (4.0)
Overall *B* factor from Wilson plot (Å^2)	40	35
$CC_{1/2}$	1.00 (0.93)	1.00 (0.91)
R_{cryst}/ R_{free} (%)	21.2/23.9	19.2/22.8
r.m.s.d 1-2 bonds (Å)	0.009	0.008
r.m.s.d 1-3 angles (°)	1.365	1.591
Avge main chain B (Å^2)	55	48
Avge side chain B (Å^2)	57	51
NADPH B (Å^2)	43	61
Avge water B (Å^2)	44	37

Section S4. Molecular Modelling.

Homology Modelling

To provide a structure including the unresolved residues, a homology model was generated. For this purpose, the Modeller[16,17] PlugIn PyMod2.0[18] was used with PyMOL 1.8.[19] For this purpose, all chains from 6TOE (NADH-IRED-Ms NAD^+ complex) were separated and superposed. NAD^+ was extracted from chain A of 6TOE and was adapted via PyMOL builder to provide a functional NADH cofactor. The atom names were adapted manually.

Generation of Substrate Complex and Parametrization

2-Methyl-1-pyrroline was used as model substrate. The structure was gained from PubChem compound database[20] and it was manually docked next to the NADPH in three different starting conformations to reduce the initial bias. Parameters for 2-methylpyrroline and NADH were calculated with antechamber.[21] Using the parameters listed in MOL2 and FRCMOD files, parameter XML files were generated. The according coordinate and parameter files are provided on the data repository of the University of Stuttgart.

Molecular Dynamics Simulation

The pKa values of the models' side chains were calculated using PROPKA[22,23] provided by PDB2PQR server (version 2.0.0).[24] A pH of 7 and the Parse forcefield were utilized. According to these results, all residues differing from the standard protonation state (D117, D171) were protonated via OpenMM Modeller class. Mutations of NADH-IRED-Ms were introduced via PDBfixer utility. The simulations were performed using OpenMM 7.4.1[25,26] utilizing NVIDIA CUDA GPU platform.[27] General Amber force field (GAFF) and Amber14 force field were used.[29,29] The cubic box with a padding of 1.5 nm was solvated with water (tip4p-Ew water model),[30] the protein charge was neutralized, and an ionic strength of 0.1 M NaCl was applied, and the total charge was neutralized. Energy minimization was performed, until 10 kJ/mole

tolerance energy. A reference temperature of 300 K and a pH of 7 were utilized and the Langevin integrator was used with a friction coefficient of 1/ps and a step size of 2 fs.[31] Additionally, the Particle Mesh Ewald method was used to compute long range Coulomb interactions with a 1 nm nonbonded cut-off for the direct space interactions. To equilibrate the solvent, a 5 ns pressure coupled equilibration with Monte Carlo barostat was performed at a pressure of 1 atm assuming the reference temperature of 300 K (temperature coupling provided by Langevin integrator). Thereby, the protein backbone and each ligand were restrained, with a force of 100 and 150 kJ/mole*Å2, respectively. To enable conformational rearrangements toward a proper substrate-binding conformation, a similar equilibration was performed for 50 ns, whereby the protein restraints were removed but the ligand restraints were kept. Successively, substrate and cofactor restrained were removed, and the equilibration was continued for 0.5 ns and 5 ns, respectively. With the resulting system, a production of 10 replicates à 50 ns was performed under periodic boundary conditions. The trajectories were written every 1000 steps.

Analysis

The analysis was mainly performed utilizing MDTraj.[32] Thereby, the 10 replicates per system were considered separately. To identify residues interacting with the imine nitrogen, residues within a sphere with a radius of 10 Å was defined around the hydride donor hydrogen of the NADH in the respective crystal structure were considered. The contact frequency (< 2.5 Å) between hydrogens of the selected binding site residues (Table S7) and the imine nitrogen were computed with a modulated code of the MDTraj-based Contact Map Explorer (https://contact-map.readthedocs.io/). To estimate the stereoselectivity, only approximated productive substrate conformations with (distance of < 3 Å between imine nitrogen + the potentially proton donating hydrogens; < 4.5 Å between imine carbon + the closer hydride of NADH cofactor) were considered. These frames were superposed on energy minimized structure to provide

comparable coordinates and the stereopreference was derived by computing the dihedral angle α formed by two planes (Figure S5). The first plane is spanned by imine nitrogen, C2 and C5 carbon of 2-methylpyrroline. The second plane is spanned by C2 and C5 carbon of 2-methylpyrroline and the closer hydride donating hydrogen of NADH. In the case of $\alpha > 0.1$ rad, the conformation was classified as *(S)*-selective and in the case of $\alpha < -0.1$ rad, as *(R)*-selective. Frames in between this range were not considered for the evaluation of the stereoselectivity.

Table S7. Atoms considered in the calculation of substrate binding site residue contact frequencies with the imine nitrogen.

NADH-IRED-Ms			(*S*)-NADH_V11	
Residue	Hydrogen atom(s)	Standard numbering	Residue	Hydrogen atom(s)
S15	HG	32	S15	HG
N65	H04+H05		N65	H04+H05
T94	HG1	110	T94	HG1
S95	HG	111	S95	HG
T123	HG2	139	T123	HG2
S132	HG	148	S132	HG
D171	HD2	187	D171	HD2
W179	HE1	195	W179	HE1
R223	H12-H15	239	R223	H12-H15
H242	HD1	257.1	Y242	HH

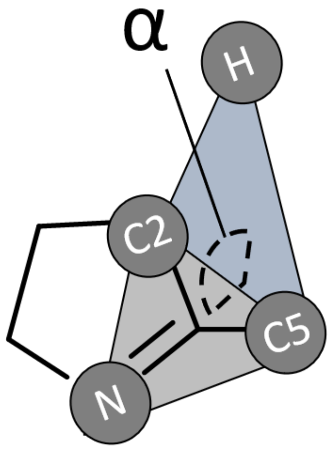

Figure S5. Scheme of the planes spanning defining dihedral angle α which serves as criterium to decide over the frames' stereopreference.

Detailed description of simulation results

While the simulation of systems NADH-IRED-Ms_1 and NADH-IRED-Ms_3 solely displayed contacts with S95 and W179, some frames of system NADH-IRED-Ms_2 did also display close contacts to the conventional proton donor D171. In the systems of (*S*)-NADH_V11, no close contacts were observed with S95. Indeed, the contact frequency with D171 and W179 was remarkably higher. Thereby, the simulations of system (*S*)-NADH_V11_1 displayed contact with both, D171 and W179, while system (*S*)-NADH_V11_2 and (*S*)-NADH_V11_3 solely showed contacts with D171 and W179, respectively.

The ratio of theoretical stereopreference was derived by trends in simulation states with close 2-methylpyrroline substrate distances towards NADH and S95, D171 and W179 side chains. Assuming D171 as being actively or at least passively involved in the protonation of the imine moiety, ternary complexes of NADH-IRED-Ms and (*S*)-NADH_V11-NADH with 2-methylpyrroline were derived from the states with the closest distances to the catalytic atoms. As the detection of a 'real' near-attack complexes in MD simulations often affords the application of complex and time-consuming enhanced sampling methods,[33-37] this focused approach settles with approximated productive binding modes.

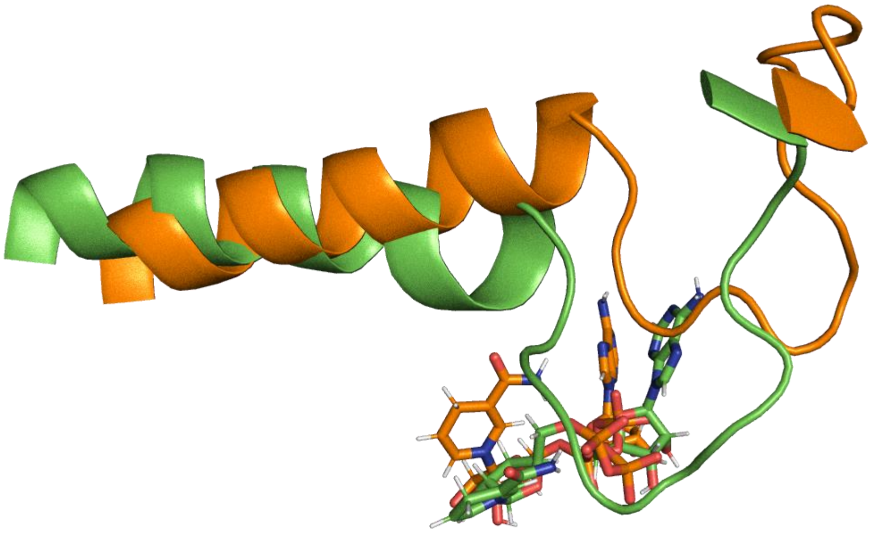

Figure S6. The five additional mutants in (*S*)-NADH_V11 (green) lead to a shifted loop-helix element which seem to alter the nicotinamide orientation of the cofactor compared to NADH-IRED-Ms (orange).

Section S5. Appendix

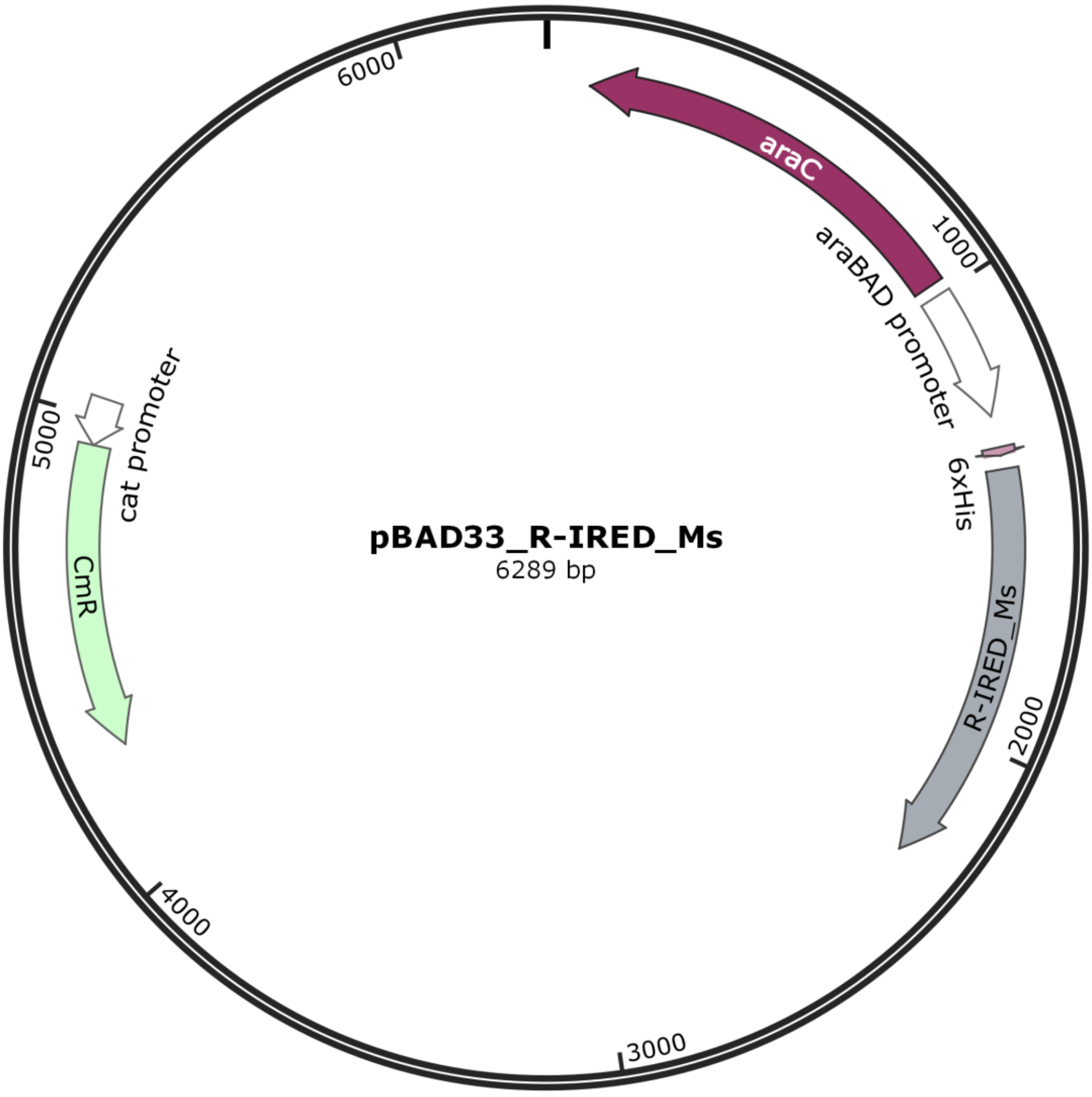

Figure S7. General plasmid map of the pBAD constructs to generate the variants with switched cofactor specificity and altered stereoselectivity.

DNA-sequence of (*R*)-IRED-*Ms*

ATGGGCAGCAGCCATCATCATCATCATCACAGCAGCGGCCTGGTGCCGCGCGGCA

GCCATATGAAACCGACCCTGACCGTTATTGGCGCTGGCCGTATGGGCTCCGCACT

GATTAAAGCATTCCTGCAATCTGGCTACACGACCACGGTGTGGAACCGTACCAAA

GCCAAAAGCGAACCGCTGGCAAAACTGGGCGCACATCTGGCTGATACGGTGCGT

GACGCCGTTAAACGCAGCGATATTATCGTGGTTAATGTGCTGGATTATGACACCT

CTGATCAGCTGCTGCGCCAAGACGAAGTGACGCGTGAACTGCGCGGCAAACTGC

TGGTTCAGCTGACCAGCGGTTCTCCGGCACTGGCTCGTGAACAGGAAACGTGGGC

GCGCCAACATGGCATTGATTATCTGGACGGTGCGATCATGGCCACCCCGGATTTT

ATTGGCCAGGCAGAATGCGCTCTGCTGTACAGTGGTTCCGCGGCCCTGTTCGAAA

AACACCGTGCTGTCCTGAATGTGCTGGGCGGTGCCACCAGCCATGTCGGCGAAGA

TGTTGGTCATGCCTCAGCACTGGACAGCGCCCTGCTGTTTCAGATGTGGGGCACC

CTGTTCGGTACGCTGCAAGCACTGGCTATTTCTCGCGCAGAAGGCATCCCGCTGG

AAAAAACCACGGCGTTTATCAAACTGACCGAACCGGTCACCCAGGGTGCCGTTGC

AGATGTCCTGACCCGTGTTCAGCAAAATCGCCTGACCGCAGACGCTCAGACGCTG

GCAAGTCTGGAAGCTCATAACGTGGCGTTCCAACACCTGCTGGCCCTGTGTGAAG

AACGTAATATCCATCGCGGTGTTGCGGATGCCATGTACTCCGTTATTCGTGAAGC

GGTCAAAGCCGGCCACGGTAAAGATGACTTTGCAATTCTGACCCGCTTCCTGAAA

TAA

Protein-sequence of (*R*)-IRED-*Ms*

HHHHHH = His_6-Tag; **M** = original starting methionine

MGSSHHHHHHSSGLVPRGSHMKPTLTVIGAGRMGSALIKAFLQSGYTTTVWNRTK

AKSEPLAKLGAHLADTVRDAVKRSDIIVVNVLDYDTSDQLLRQDEVTRELRGKLLVQ

LTSGSPALAREQETWARQHGIDYLDGAIMATPDFIGQAECALLYSGSAALFEKHRAV

LNVLGGATSHVGEDVGHASALDSALLFQMWGTLFGTLQALAISRAEGIPLEKTTAFI

KLTEPVTQGAVADVLTRVQQNRLTADAQTLASLEAHNVAFQHLLALCEERNIHRGV

ADAMYSVIREAVKAGHGKDDFAILTRFLK

Protein-sequence of NADH-IRED-Ms

HHHHHH = His_6-Tag; M = original starting methionine; X = point mutation

MGSSHHHHHHSSGLVPRGSHMKPTLTVIGAGRMGSALIKAFLQSGYTTTVWEYEKA
RSEPLAKLGAHLADTVRDAVKRSDIIVVNVIDYDVSDQLLRQDEVTRELRGKLLVQL
TSGSPALAREQETWARQHGIDYLDGAIMATPDFIGQAECALLYSGSAALFEKHRAVL
NVLGGATSHVGEDVGHASALDSALLFQMWGTLFGTLQALAISRAEGIPLEKTTAFIK
LTEPVTQGAVADVLTRVQQNRLTADAQTLASLEAHNVAFQHLLALCEERNIHRGVA
DAMYSVIREAVKAGHGKDDFAILTRFLK

Protein-sequence of (*S*)-NADH_V11

HHHHHH = His_6-Tag; M = original starting methionine; X = point mutation

MGSSHHHHHHSSGLVPRGSHMKPTLTVIGAGRMGSALIKAFLQSGYTTTVWEYEKA
RSEPLAKLGAHLADTVRDAVKRSDIIVVNVIDYDVSDQLLRQDEVTRELRGKLLVQL
TSGSPALAREQETWARQHGIDYLDGAIMATPDFIGQAECALLYSGSAALFEKHRAVL
NVLGGATSHVGEDVGHASALDSALLFQMWGTLFGTLQALAISRAEGIPLEKTTAFIK
LTEPVTQGAVADVLTRVQQNRLTADAQTLASLVYDYLFQHLLALCEERNIHRGVAD
AMYSVIREAVKAGHGKDDFAILTRFLK

References.

(1) Fademrecht, S.; Scheller, P. N.; Nestl, B. M.; Hauer, B.; Pleiss, J. Identification of Imine Reductase-Specific Sequence Motifs. *Proteins Struct. Funct. Bioinforma.* **2016**, *84*, 600–610.

(2) Velikogne, S.; Resch, V.; Dertnig, C.; Schrittwieser, J. H.; Kroutil, W. Sequence-Based In-Silico Discovery, Characterisation, and Biocatalytic Application of a Set of Imine Reductases. *ChemCatChem* **2018**, *10*, 3236–3246.

(3) Borlinghaus, N.; Nestl, B. M. Switching the Cofactor Specificity of an Imine Reductase. *ChemCatChem* **2018**, *10*, 183-187.

(4) Scheller, P. N.; Nestl, B. M. The biochemical characterization of three imine-reducing enzymes from *Streptosporangium roseum* DSM43021, *Streptomyces turgidiscabies* and *Paenibacillus elgii. Appl. Microbiol. Biotechnol.* **2016**, *100*, 10509-10520.

(5) Braman, J.; Papworth, C.; Greener, A. Site-Directed Mutagenesis Using Double-Stranded Plasmid DNA Templates. In *In Vitro Mutagenesis Protocols*; Trower, M. K., Ed.; Humana Press: Totowa, NJ, 1996; pp 31–44.

(6) Gibson, D. G.; Young, L.; Chuang, R. Y.; Venter, J. C.; Hutchison, C. A.; Smith, H. O. Enzymatic Assembly of DNA Molecules up to Several Hundred Kilobases. *Nat. Methods* **2009**, *6*, 343-345.

(7) Kille, S.; Acevedo-Rocha, C. G.; Parra, L. P.; Zhang, Z. G.; Opperman, D. J.; Reetz, M. T.; Acevedo, J. P. Reducing Codon Redundancy and Screening Effort of Combinatorial Protein Libraries Created by Saturation Mutagenesis. *ACS Synth. Biol.* **2013**, *2*, 83-92.

(8) Kabsch, W. *XDS. Acta Crystallogr. Sect. D Biol. Crystallogr.* **2010**, *D66*, 125-132.

(9) Evans, P. Scaling and Assessment of Data Quality. In *Acta Crystallographica Section D: Biological Crystallography*; **2006**, *62*, 72-82.

(10) Winter, G. Xia2: An Expert System for Macromolecular Crystallography Data Reduction. *J. Appl. Crystallogr.* **2010**, *43*, 186-190.

(11) Vagin, A.; Teplyakov, A. MOLREP: An Automated Program for Molecular Replacement. *J. Appl. Crystallogr.* **1997**, *30*, 1022-1025.

(12) Vagin, A.; Teplyakov, A. Molecular Replacement with MOLREP. *Acta Crystallogr. Sect. D Biol. Crystallogr.* **2010**, *66*, 22-25.

(13) Sharma, M.; Mangas-Sanchez, J.; France, S. P.; Aleku, G. A.; Montgomery, S. L.; Ramsden, J. I.; Turner, N. J.; Grogan, G. A Mechanism for Reductive Amination Catalyzed by Fungal Reductive Aminases. *ACS Catal.* **2018**, *8*, 11534-11541.

(14) Emsley, P.; Cowtan, K. Coot: Model-Building Tools for Molecular Graphics. *Acta Crystallogr. Sect. D Biol. Crystallogr.* **2004**, *60*, 2126-2132.

(15) Murshudov, G. N.; Vagin, A. A.; Dodson, E. J. Refinement of Macromolecular Structures by the Maximum-Likelihood Method. *Acta Crystallographica Section D: Biological Crystallography*. **1997**, *53*, 240-255.

(16) Sali, A., Blundell, T. L. Comparative Protein Modelling by Satisfaction of Spatical

Restraints, *J. Mol. Biol.* **1993**, *234,* 779-815.

(17) Webb, B.; Sali, A. Comparative Protein Structure Modeling Using MODELLER. *Curr. Protoc. Bioinforma.* **2016**, *54,* 5.6.1-5.6.37

(18) Janson, G.; Zhang, C.; Prado, M. G.; Paiardini, A. PyMod 2.0: Improvements in Protein Sequence-Structure Analysis and Homology Modeling within PyMOL. *Bioinformatics* **2017**, *33*, 444-446.

(19) Schrödinger, LLC. *The PyMOL Molecular Graphics System, Version 1.8*; 2015.

(20) Kim, S.; Thiessen, P. A.; Bolton, E. E.; Chen, J.; Fu, G.; Gindulyte, A.; Han, L.; He, J.; He, S.; Shoemaker, B. A.; Wang, J.; Yu, B.; Zhang, J.; Bryant, S. H. PubChem Substance and Compound Databases. *Nucleic Acids Res.* **2016**, *44*, D1202-D1213.

(21) Wang, J.; Wang, W.; Kollman, P. A; Case, D. A. Antechamber, An Accessory Software Package For Molecular Mechanical Calculations. *J. Am. Chem. Soc* **2001**, *222,* U403.

(22) Søndergaard, C. R.; Olsson, M. H. M.; Rostkowski, M.; Jensen, J. H. Improved Treatment of Ligands and Coupling Effects in Empirical Calculation and Rationalization of p K a Values. *J. Chem. Theory Comput.* **2011**, *7,* 2284-2295.

(23) Olsson, M. H. M.; Søndergaard, C. R.; Rostkowski, M.; Jensen, J. H. PROPKA3: Consistent Treatment of Internal and Surface Residues in Empirical PKa Predictions BT - Journal of Chemical Theory and Computation. *J. Chem. Theory Comput.* **2011**, *7,* 525-537.

(24) Dolinsky, T. J.; Nielsen, J. E.; McCammon, J. A.; Baker, N. A. PDB2PQR: An Automated Pipeline for the Setup of Poisson-Boltzmann Electrostatics Calculations. *Nucleic Acids Res.* **2004**, *32*, W665-W667.

(25) Eastman, P.; Pande, V. S. OpenMM: A Hardware-Independent Framework for Molecular Simulations. *Comput. Sci. Eng.* **2010**, *12,* 34-39.

(26) Eastman, P.; Swails, J.; Chodera, J. D.; McGibbon, R. T.; Zhao, Y.; Beauchamp, K. A.; Wang, L. P.; Simmonett, A. C.; Harrigan, M. P.; Stern, C. D.; Wiewiora, R. P.; Brooks, B. R.; Pande, V. S. OpenMM 7: Rapid Development of High Performance Algorithms for Molecular Dynamics. *PLoS Comput. Biol.* **2017**, *13,* e1005659.

(27) Cook, S. (2012). *CUDA Programming: A Developer's Guide to Parallel Computing with GPUs* (1st ed.). San Francisco, CA, USA: Morgan Kaufmann Publishers Inc.

(28) Case, D. A.; Babin, V.; Berryman, J. T.; Betz, R. M.; Cai, Q.; Cerutti, D. S.; Cheatham III, T. E.; Darden, T. A.; Duke, R. E.; Gohlke, H.; Goetz, A. W.; Gusarov, S.; Homeyer, N.; Janowski, P.; Kaus, J.; Kolossváry, I.; Kovalenko, A.; Lee, T. S.; LeGrand, S.; Luchko, T.; Luo, R.; Madej, B.; Merz, K. M.; Paesani, F.; Roe, D. R.; Roitberg, A.; Sagui, C.; Salomen-Ferrer, R.; Seabra, G.; Simmerling, G. L.; Smith, W.; Swails, J.; Walker, R. C.; Wang, J.; Wolf, R. M.; Wu, X.; Kollman, P. A. AMBER14. *AMBER 14*. 2014.

(29) Wang, J.; Wolf, R. M.; Caldwell, J. W.; Kollman, P. A.; Case, D. A. Development and Testing of a General Amber Force Field. *J. Comput. Chem.* **2004**, *25,* 1157-1174.

(30) Horn, H. W.; Swope, W. C.; Pitera, J. W.; Madura, J. D.; Dick, T. J.; Hura, G. L.;

Head-Gordon, T. Development of an Improved Four-Site Water Model for Biomolecular Simulations: TIP4P-Ew. *J. Chem. Phys.* **2004**, *120*, 9665-9678.

(31) Bussi, G.; Parrinello, M. Accurate Sampling Using Langevin Dynamics. *Phys. Rev. E - Stat. Nonlinear, Soft Matter Phys.* **2007**, *75*, 056707.

(32) McGibbon, R. T.; Beauchamp, K. A.; Harrigan, M. P.; Klein, C.; Swails, J. M.; Hernández, C. X.; Schwantes, C. R.; Wang, L. P.; Lane, T. J.; Pande, V. S. MDTraj: A Modern Open Library for the Analysis of Molecular Dynamics Trajectories. *Biophys. J.* **2015**, *109*, 1528-1532.

(33) Carter, E. A.; Ciccotti, G.; Hynes, J. T.; Kapral, R. Constrained Reaction Coordinate Dynamics for the Simulation of Rare Events. *Chem. Phys. Lett.* **1989**, *156*, 472-477.

(34) Yang, L.; Liu, C. E.; Shao, Q.; Zhang, J.; Gao, Y. Q. From Thermodynamics to Kinetics: Enhanced Sampling of Rare Events. *Acc. Chem. Res.* **2015**, *48*, 947-955.

(35) Fishelovitch, D.; Shaik, S.; Wolfson, H. J.; Nussinov, R. Theoretical Characterization of Substrate Access/Exit Channels in the Human Cytochrome P450 3A4 Enzyme: Involvement of Phenylalanine Residues in the Gating Mechanism. *J. Phys. Chem. B* **2009**, *113*, 13018-13025.

(36) Hamelberg, D.; Mongan, J.; McCammon, J. A. Accelerated Molecular Dynamics: A Promising and Efficient Simulation Method for Biomolecules. *J. Chem. Phys.* **2004**, *120, 11919-11929.*

(37) Tiwary, P.; van de Walle, A. A Review of Enhanced Sampling Approaches for Accelerated Molecular Dynamics. In *Springer Series in Materials Science*; Weinberger C. R.; Tucker, G. J., Ed., Springer Link, Switzerland, 2016, pp195-221.
